# Management of antipsychotic-related sexual dysfunction

**DOI:** 10.1192/j.eurpsy.2022.1836

**Published:** 2022-09-01

**Authors:** C. Rodrigues

**Affiliations:** Centro Hospitalar Psiquiátrico de Lisboa, Serviço De Reabilitação, Lisboa, Portugal

**Keywords:** sexual, Treatment, dysfunction, antipsychotic

## Abstract

**Introduction:**

Sexual dysfunction (SD) can often be a side-effect of treatment with antipsychotics (APS). It often jeopardizes long-term adherence to treatment, while deeply affecting the patient’s quality of life. The pathogenic mechanisms may be associated with postsynaptic dopamine antagonism, a_1_-antagonism and prolactin elevation. APS-induced hyperprolactinemia has been linked to the occurrence of galactorrhea, gynecomastia, amenorrhea and SD.

**Objectives:**

To synthesize the available evidence on the management of APS-related sexual dysfunction, with a main focus on the second-generation antipsychotics.

**Methods:**

A search for randomized controlled trials (RCT) published between 2021 and 2011 on PubMed was made using the keywords “sexual”; “dysfunction”; “antipsychotic” and “treatment”, from which resulted sixteen articles. Only six of those were considered relevant for the study’s objectives.

**Results:**

Three studies focused on the comparison between different APS and prolactin levels and SD occurrence, showing that treatment with aripiprazole is mostly related to prolactin levels with the normal range and a lower incidence of sexual dysfunction. Addition of aripiprazole to previous APS may be associated with normalization of sexual function and pose as a possible management option. Adjunctive treatment with tadalafil showed no significant effect on its primary outcome.

**Conclusions:**

There seems to be a general consensus that patients treated with first-generation antipsychotics (FGA), along with risperidone, paliperidone and amissulpride show higher prolactin levels and incidence of SD. Whether there is a causal relationship between these two variables still remains a question. Larger and more prolonged trials are still needed to evaluate APS-related sexual dysfunction and its management.

**Disclosure:**

No significant relationships.

